# Investigation of Recessed Gate AlGaN/GaN MIS-HEMTs with Double AlGaN Barrier Designs toward an Enhancement-Mode Characteristic

**DOI:** 10.3390/mi11020163

**Published:** 2020-02-03

**Authors:** Tian-Li Wu, Shun-Wei Tang, Hong-Jia Jiang

**Affiliations:** International College of Semiconductor Technology, National Chiao Tung University, Hsinchu 30010, Taiwan; tangandy.icst07g@nctu.edu.tw (S.-W.T.);

**Keywords:** GaN, metal-insulator-semiconductor high-electron-mobility transistor (MIS-HEMT), recessed gate, double barrier

## Abstract

In this work, recessed gate AlGaN/GaN metal-insulator-semiconductor high-electron-mobility transistors (MIS-HEMTs) with double AlGaN barrier designs are fabricated and investigated. Two different recessed depths are designed, leading to a 5 nm and a 3 nm remaining bottom AlGaN barrier under the gate region, and two different Al% (15% and 20%) in the bottom AlGaN barriers are designed. First of all, a double hump trans-conductance (*g*_m_)–gate voltage (*V*_G_) characteristic is observed in a recessed gate AlGaN/GaN MIS-HEMT with a 5 nm remaining bottom Al_0.2_Ga_0.8_N barrier under the gate region. Secondly, a physical model is proposed to explain this double channel characteristic by means of a formation of a top channel below the gate dielectric under a positive *V*_G_. Finally, the impacts of Al% content (15% and 20%) in the bottom AlGaN barrier and 5 nm/3 nm remaining bottom AlGaN barriers under the gate region are studied in detail, indicating that lowering Al% content in the bottom can increase the threshold voltage (*V*_TH_) toward an enhancement-mode characteristic.

## 1. Introduction

AlGaN/GaN high-electron-mobility transistors (HEMTs) are promising for power switching applications due to the wide band gap, large breakdown electric field, and the inherent high electron mobility due to two-dimensional electron gas (2DEG) [1,2]. Normally, conventional AlGaN/GaN Schottky HEMTs suffer high gate leakage, resulting in an unfavorable power loss during an off-state condition and a low gate overdrive during an on-state condition. In order to tackle this issue, Metal-insulator-semiconductors high electrons mobility transistors (MIS-HEMTs) have gained attentions recently [2,3,4]. Inserting a dielectric in the interface between AlGaN and gate metal significantly reduces the gate leakage current, allowing a high gate overdrive to have a fast switch from off-state to on-state operation. Due to a piezoelectric and polarization effects, a two dimensional electron gas (2DEG) is naturally formed in the interface between GaN and AlGaN, leading to depletion mode (*V*_TH_<0) characteristics. However, an enhancement mode characteristic is more favorable in practical applications due to a lower power consumption, less failure issues, and a flexible integration. So far, there are several approaches to realize an enhancement mode operation, such as a recessed gate structure [5,6,7], p-GaN/p-AlGaN gate [2,8], Fluoride-based plasma treatment [9], the metal–oxide–semiconductor field-effect transistor structure [10], cascode-based topology in connecting high voltage D-mode HEMTs with a low voltage Si MOSFETs or E-mode HEMTs [11,12], etc. HEMTs with a p-GaN/p-AlGaN gate generally suffer the challenges to effectively dope the Mg into top GaN or AlGaN layer and to remove the top p-GaN/p-AlGaN layer in the access region. The thermal stability of the charges induced by Fluoride-based plasma remains a challenge. The MOS-type GaN FETs suffer the low electron mobility due to the disappearance of the 2DEG. The cascode-based topology with dual GaN-based transistors increase the active area and complicates the layout designs. Therefore, the recessed gate-based HEMT is one of the most popular architectures to obtaining an enhancement-mode characteristic because the 2DEG can be reduced under the gate by using a simple etching process, i.e., Reactive-ion etching (RIE)-based etching or atomic layer etching (ALE). Recently, the recessed gate AlGaN/GaN-based devices show a promising performance toward an enhancement-mode characteristic [5,6,7].

Typically, AlGaN/GaN-based HEMTs are fabricated with the single AlGaN barrier layer. A multi barrier device was demonstrated first in GaAs-based devices in 1985 [13]. Since 1999, a depletion mode double AlGaN barrier design was first demonstrated by Gaska et al. [14]. Afterwards, depletion-mode double AlGaN barrier HEMTs have been explored in details in [15,16,17,18], showing a high current drive due to a second transconductance (gm) and lower access resistance. Furthermore, the recent demonstration using AlGaN/AlN/GaN/AlN epitaxy stack to achieve the double channel has attracted a lot of attentions [19]. Although the demonstration of the GaN-based HEMTs with a double barrier exhibits the promising characteristics, the impacts of the Al% in the top and bottom AlGaN barriers are still unclear. Furthermore, the investigation of the combination of the recessed-based approach in the HEMTs with double AlGaN barrier designs is lacking as well, which can further provide the insightful analysis to understand the device physics and structure designs toward an enhancement-mode characteristic.

In this work, recessed gate MIS-HEMTs with double AlGaN barrier designs with different recessed depths (3 nm or 5 nm remaining bottom AlGaN barrier under the gate region) and different Al% content in the bottom AlGaN barrier (15% and 20%) are fabricated and investigated. First of all, we observed a double hump *g*_m_–*V*_G_ characteristic in a recessed gate AlGaN/GaN MIS-HEMT with a 5nm remaining bottom Al_0.2_Ga_0.8_N barrier under the gate region. Then, a physical model considering the formation of the top channel under a positive *V*_G_ is proposed to explain this double hump in the *g*_m_–*V*_G_ characteristic. Furthermore, the impacts from the Al% (20% and 15%) in bottom AlGaN barrier and recessed depth (3 nm and 5 nm remaining bottom AlGaN barrier under the gate region) are discussed to understand the device characteristics and the V_TH_ can be increased by designing the device with a lower Al% in the bottom AlGaN barrier.

## 2. Device Fabrications

**Figure 1:**
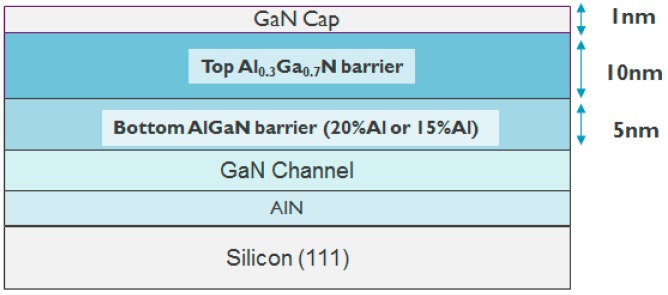
Schematic of the epitaxy structure with a double AlGaN barrier used in this study.

**Figure 2:**
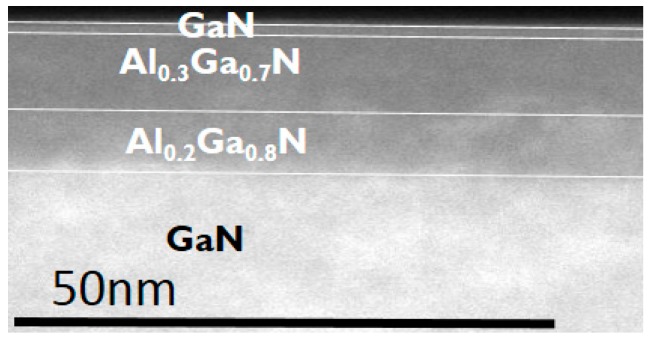
Transmission electron microscopy (TEM) images of the epitaxy structure with a double AlGaN barrier.

**Figure 3:**
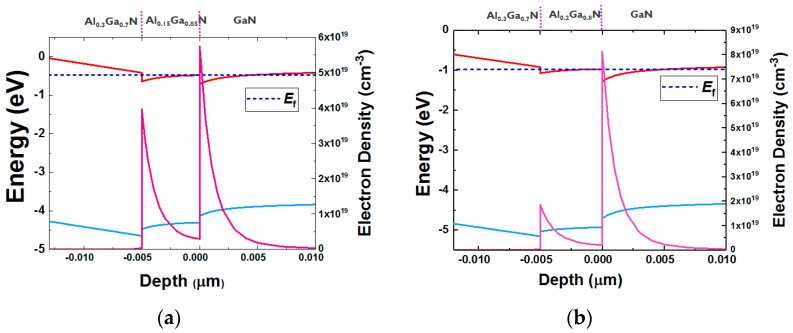
Simulated band diagrams with an Al_0.3_Ga_0.7_N/Al_0.15_Ga_0.85_N barrier (**a**) and an Al_0.3_Ga_0.7_N/Al_0.2_Ga_0.8_N barrier (**b**).

**Figure 4:**
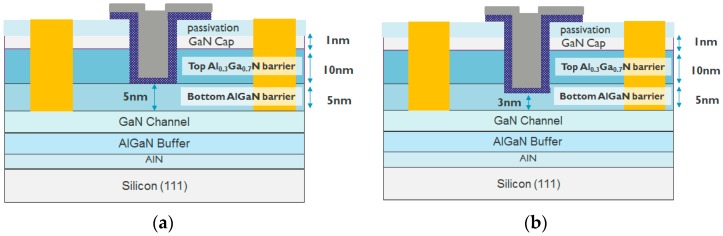
Schematic of device structures of recessed gate AlGaN/GaN MIS-HEMTs with a 5 nm remaining bottom AlGaN barrier under the gate region (**a**) and a 3 nm remaining bottom AlGaN barrier under the gate region (**b**).

Figure 1 shows the schematic of the epitaxy structure in this study and Figure 2 shows an example of the transmission electron microscopy (TEM) image in an epitaxy structure with a double AlGaN barrier. This structure was grown by metal–organic chemical vapor deposition (MOCVD) on a silicon (111) substrate and consists of an AlN nucleation layer, a GaN channel, a bottom AlGaN barrier with two different Al contents (20% and 15%), a top AlGaN barrier with 30% Al content, and a 1 nm GaN cap. The Al% (20% and 30%) in AlGaN barrier is calibrated with the XPS by using a single AlGaN barrier hetero-structure. The calibrated growing conditions in MOCVD are used for the double AlGaN barrier hetero-structure. Figure 3 shows the simulated band diagram with the double AlGaN barriers, clearly indicating the existence of the electrons in the interface between top AlGaN/bottom AlGaN and bottom AlGaN/GaN. The recessed gate structure was formed by reactive ion etching (RIE). In order to control the etching depth, low etching rate of 5Å/sec is achieved by the mixed BCl_3_ (10 sccm)/Cl_2_ (15 sccm) gas. A gate recessed process is performed and a 15-nm Plasma-enhanced chemical vapor deposition (PECVD) Si_3_N_4_ is deposited as a surface passivation layer in the access region and a gate dielectric. TiN is used as the gate metal. The Ti/Al-based Au-free Ohmic contacts were formed by etching the Si_3_N_4_ layers and etching the AlGaN barrier. This was followed by annealing at 600 °C for 1 min in N_2_, resulting in 1 ohm.mm of R_c_ (contact resistance). Figure 4 shows the schematic of recessed gate MIS-HEMTs with a double AlGaN barrier. The important varied parameters are summarized in Table 1. The devices with *L*g = 1 um, *L*gs = 2 um, and *L*gd = 6 um are fabricated for electrical characterizations.

## 3. Results

**Figure 5:**
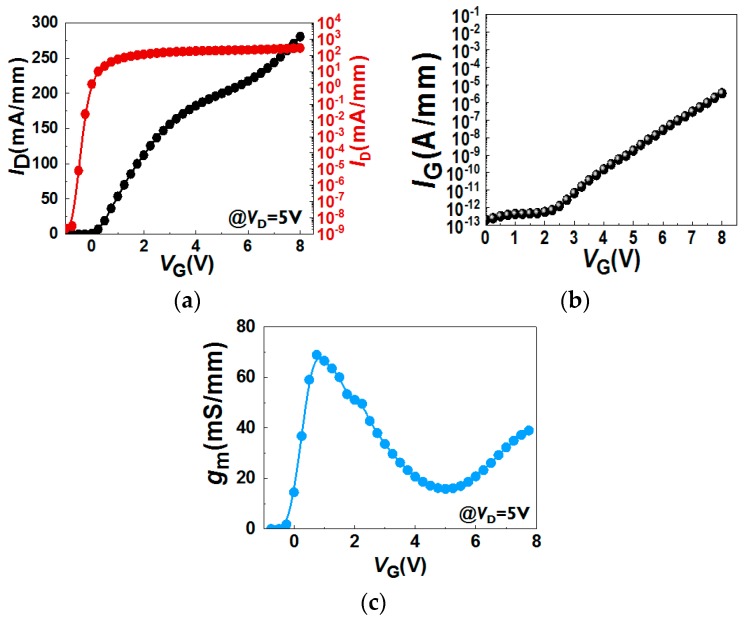
*I*_D_–*V*_G_ (**a**), *I*_G_–*V*_G_ (**b**) and *g*_m_–*V*_G_ (**c**) characteristics in a recessed gate MIS-HEMTs with a 5nm remaining bottom Al_0.2_Ga_0.8_N barrier under the gate. By designing a 5 nm bottom AlGaN barrier with 20% Al content, *V*_TH_ ~0 V is realized (*V*_TH_ is defined at *V*_G_ of *I*_D_ = 0.1 mA/mm).

**Figure 6:**
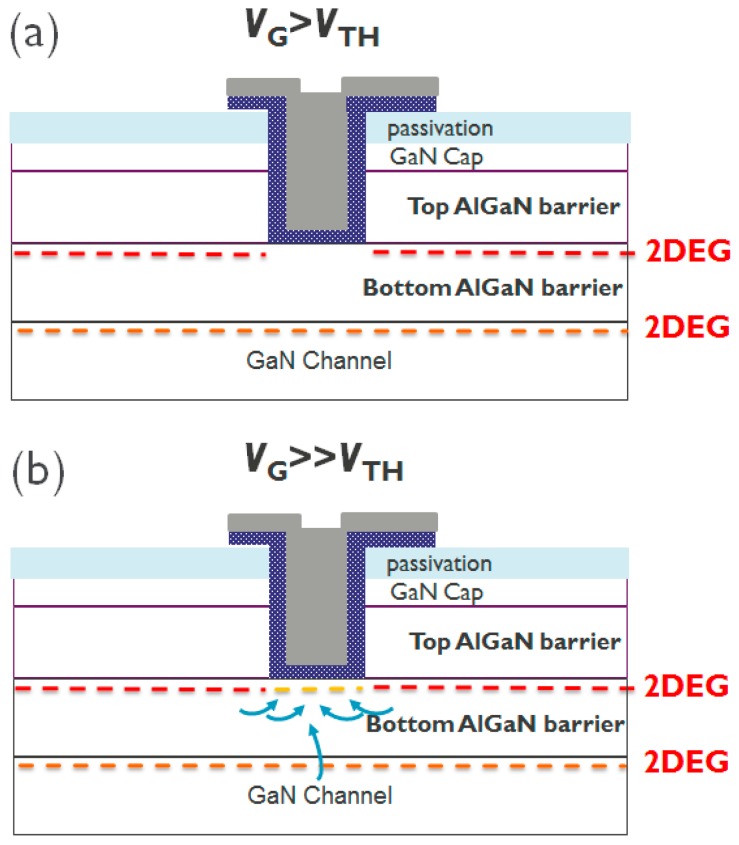
The schematic of proposed double channel model (**a**) when *V*_G_ > *V*_TH_ and (**b**) *V*_G_ >>> *V*_TH_.

**Figure 7:**
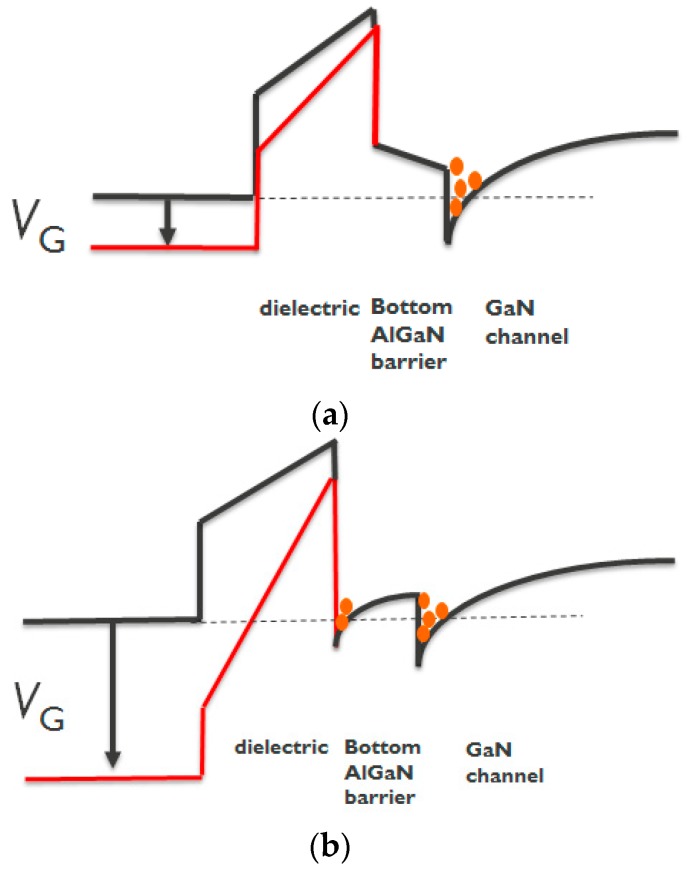
Schematic of the band diagram under (**a**) *V*_G_ > *V*_TH_ and (**b**) *V*_G_ >> *V*_TH_.

In the case of devices with a 5 nm remaining bottom AlGaN barrier under the gate area, a gate recess process is performed to etch until it reaches the surface of the bottom AlGaN barrier (Figure 4a). The *I*_D_–*V*_G_, *I*_G_-*V*_G_, and *g*_m_–*V*_G_ characteristics are shown in Figure 5. Note that all *I*_D_–*V*_G_ characteristics in this work are measured from a lower *V*_G_ till a higher *V*_G_. A double hump of *g*_m_–*V*_G_ characteristic is observed in Figure 5c, which is similar to the literature [15,16,17,18]. The double hump of *g*_m_–*V*_G_ characteristics in these references [15,16,17,18] arises from a shrinking of the depletion region below the gate, due to the depletion-mode characteristics. In our case, the recessed gate AlGaN/GaN MIS-HEMTs has a double barrier design. A double channel model that considers the electron transfer from the bottom channel to the top channel is proposed to explain the double hump *g*_m_-*V*_G_ characteristics. First of all, once the *V*_G_ is larger than *V*_TH_, the bottom channel is gradually turned on (Figure 6a and Figure 7a), resulting in a first g_m_ peak as shown in Figure 5c. It is worth noting that at this stage the top channel from source to drain is initially disconnected below the gate dielectric due to a recessed gate process. However, when the *V*_G_ is above 5 V, the top channel could be connected again, as shown in Figure 6b. In this scenario, the electrons can be transferred from the lateral 2DEG channel in the access region and/or interface below the bottom AlGaN barrier to the interface between the dielectric and the bottom AlGaN barrier [20,21] (Figure 7b). Then, the electrons can be accumulated under the gate dielectric [20,22]. This leads to the formation of the second channel under the gate, further connecting the source and drain to form the top channel leading to a second g_m_ peak (Figure 5c).

**Figure 8:**
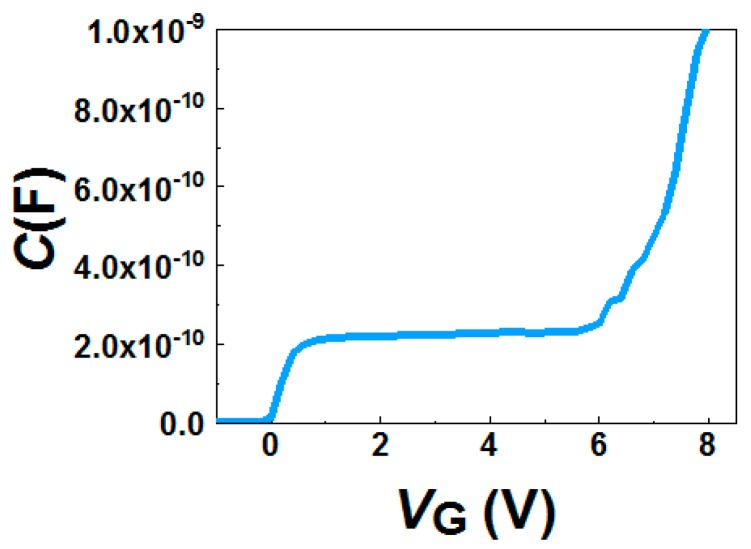
Capacitance-Voltage (CV) measurement in the device with 3 nm remaining bottom Al_0.15_Ga_0.85_N barrier.

Figure 8 shows an example of Capacitance-Voltage (CV) measurement in the device with 3 nm remaining bottom Al_0.15_Ga_0.85_N barrier. The capacitance is increase when the gate voltage is larger than 0 V, indicating the formation of the first channel (Figure 6a and Figure 7a). Once the gate voltage is applied larger enough, the capacitance is increased again, which is mainly due to the formation of the channel between dielectric and bottom AlGaN barrier (Figure 6b and Figure 7b), consistent with the reported literature [19].

**Figure 9:**
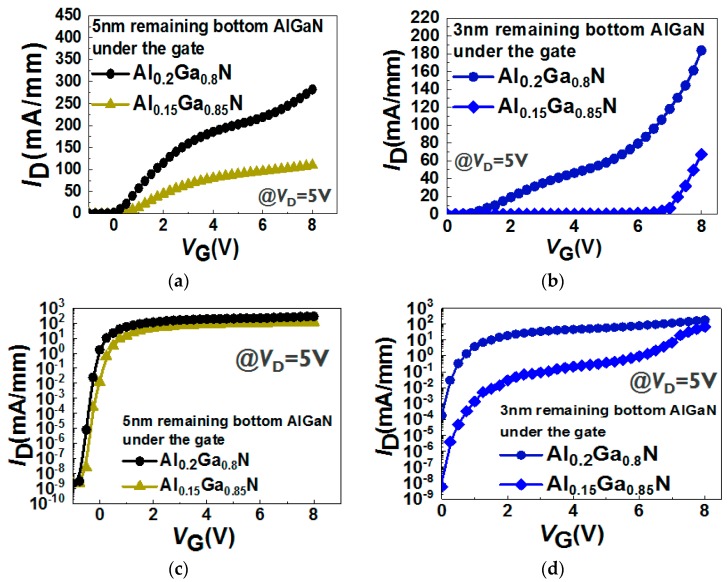
*I*_D_-*V*_G_ in a linear scale (**a**,**b**) and a logarithmic scale (**c**,**d**) in the devices with 5 nm or 3 nm remaining bottom AlGaN layer under the gate region with different Al% in the bottom AlGaN barrier.

Figure 9 shows the *I*_D_–*V*_G_ characteristics in the devices with different Al% content (Al_0.2_Ga_0.8_N and Al_0.15_Ga_0.85_N) in the bottom barriers and two different recessed depths (5 nm and 3 nm bottom AlGaN thickness under the gate area). The *I*_D_ decreases but the *V*_TH_ increases with a thinner remaining bottom AlGaN barrier (Figure 9b). Furthermore, the subthreshold slope (SS) is increased once the Al% in the bottom AlGaN barrier is decreased, which is mainly due to the low electron density in the channel between bottom AlGaN/GaN. By designing with 3nm remaining bottom Al_0.15_Ga_0.85_N barrier under the gate region, *V*_TH_ ~3.25 V is achieved (*V*_TH_ is defined at *V*_G_ of *I*_D_ = 0.1 mA/mm).

**Figure 10:**
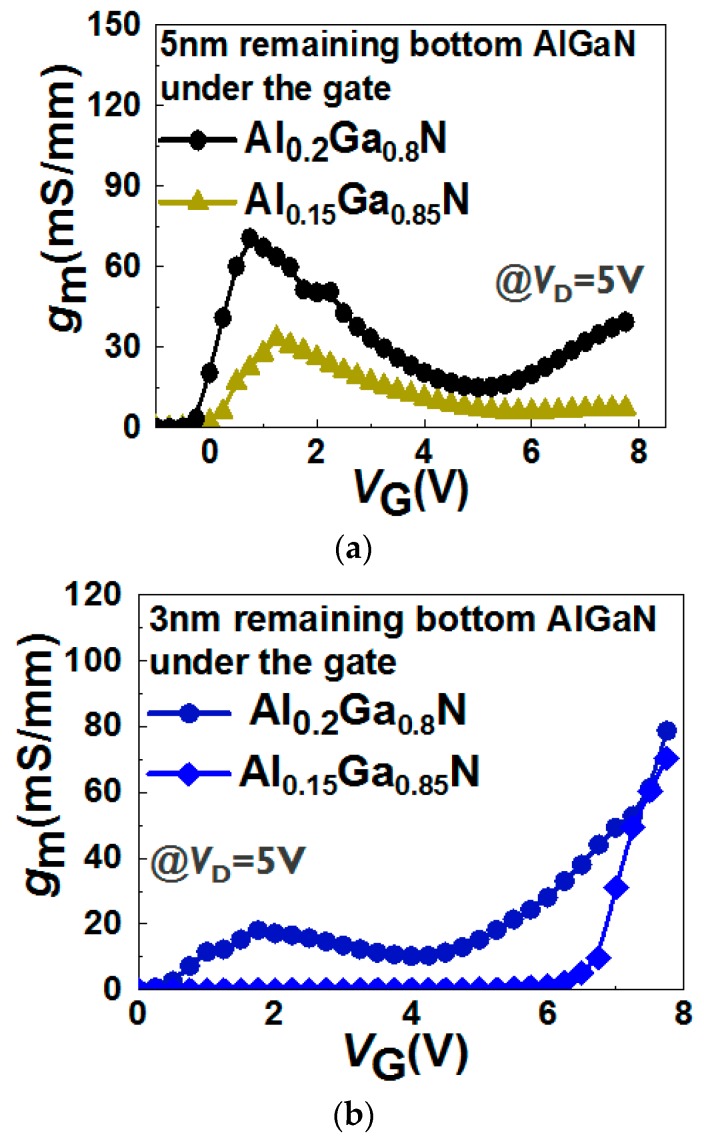
*g*_m_–*V*_G_ characteristics in the devices with a (**a**) 5 nm or (**b**) 3 nm remaining bottom AlGaN layer under the gate region with different Al% in the bottom AlGaN barrier.

Figure 10 shows the *g*_m_–*V*_G_ characteristics in the devices with different Al% content (Al_0.2_Ga_0.8_N and Al_0.15_Ga_0.85_N) in the bottom barriers and two different recessed depths (5 nm and 3 nm bottom AlGaN thickness under the gate area). In the case of the devices with a 5 nm remaining bottom AlGaN barrier, lowering the Al% in the bottom AlGaN barrier decreases the *g*_m_ peaks (Figure 10a). Furthermore, double hump *g*_m_–*V*_G_ characteristics can still be observed in the device with a 3 nm remaining bottom Al_0.2_Ga_0.8_N barrier under the gate region. First, the first *g*_m_ peak decreases with a thinner remaining bottom AlGaN barrier under the gate dielectric, suggesting that the remaining bottom AlGaN barrier under the gate region limits the current contribution from the bottom channel. Second, the *g*_m_ increases after 4 V in the device with a 3 nm remaining bottom Al_0.2_Ga_0.8_N barrier under the gate region (Figure 10b). Whereas, the *g*_m_ increases after 5 V in the devices with a 5 nm remaining bottom Al_0.2_Ga_0.8_N barrier under the gate region (Figure 10a). This is in agreement with the model proposed above: Due to a thinner AlGaN barrier, a lower gate voltage can allow the electrons to transfer from the bottom channel to the area below the gate. Third, the second *g*_m_ is higher than the first *g*_m_ in the devices with a 3 nm remaining bottom AlGaN barrier under the gate region (Figure 10b). However, the first *g*_m_ is higher than the second *g*_m_ in the devices with a 5 nm remaining bottom AlGaN barrier under the gate region (Figure 10a). These observations suggest that the main current contribution in the devices with a 5 nm remaining bottom AlGaN barrier under the gate region is the bottom channel. However, in the devices with a 3 nm remaining bottom AlGaN barrier under the gate region, the main current contribution is derived from the top channel.

Table 2 summarizes the comparisons of this work with other recent reports in double channel HEMTs, indicating our work shows the promising characteristics in terms of *I*on/*I*off ratio and *V*_TH_ toward an enhancement mode characteristic.

## 4. Conclusions

In summary, recessed gate AlGaN/GaN MIS-HEMTs with double AlGaN barrier designs are investigated and discussed. A double hump of the *g*_m_–*V*_G_ characteristic can be observed in the recessed gate AlGaN/GaN MIS-HEMTs with double AlGaN barrier designs. A physical model is proposed to explain the double channel characteristics, which is mainly due to the formation of the top channel under a high *V*_G_ bias. Once the gate voltage is applied at a high enough level, the top channel is formed, leading to an increase in drain current due to the current contribution from the top channel. Furthermore, by lowering the Al% in the bottom AlGaN barrier, the devices show a more positive *V*_TH_ with the same recessed depth, indicating that a double AlGaN barrier design in recessed gate MIS-HEMTs can be an alternative strategy to achieve an enhancement mode characteristic.

## Figures and Tables

**Table 1:** Summary of the Varied Parameters.

**Parameters**	**Top AlGaN Barrier**	**Bottom AlGaN Barrier**
Thickness	10 nm	5 nm
Al%	30%	20%
		15%
**Recessed depth**		
Remaining bottom AlGaN barrier under the gate region	–	5 nm
		3 nm

**Table 2:** Reference for the double channel GaN HEMTs.

Reference	Barrier designs	*I*_on_/*I*_off_	SS (mV/dec)	*V*_TH_ (V) @0.1mA/mm	1^nd^ *g*_m_ (mS/mm)	2^nd^ *g*_m_ (mS/mm)
**Our work**	Al_0.3_Ga_0.7_N/Al_0.2_Ga_0.8_N/GaN 5nm remaining bottom Al_0.2_Ga_0.8_N	1.2 × 10^11^	80.7	~0	68.9	39
	Al_0.3_Ga_0.7_N/Al_0.15_Ga_0.85_N/GaN 5nm remaining bottom Al_0.15_Ga_0.85_N	4.8 × 10^10^	87.9	0.25	34.8	7.6
	Al_0.3_Ga_0.7_N/ Al_0.2_Ga_0.8_N /GaN 3nm remaining bottom Al_0.2_Ga_0.8_N	-	153.1	0.5	18.8	90
	Al_0.3_Ga_0.7_N/ Al_0.15_Ga_0.85_N /GaN 3nm remaining bottom Al_0.15_Ga_0.85_N	5.5 × 10^10^	229.3	3.25	-	71.2
**Kamath et al. [17]**	Al_0.3_Ga_0.7_N/GaN/Al_0.15_Ga_0.75_N/GaN	-	-	~−7.8	34	89
**Wei et al. [19]**	AlGaN/AlN/GaN/AlN with 1.5 nm over-etch upper GaN layer	~10^9^	72	0.5	170	103
**Lee et al. [23]**	AlGaN/GaN/AlGaN/GaN Fin Structure	-	-	0.2	133	-
**Deen et al. [24]**	AlN/GaN/AlN/GaN	-	-	~−4	190	-
